# *Edin* Expression in the Fat Body Is Required in the Defense Against Parasitic Wasps in *Drosophila melanogaster*

**DOI:** 10.1371/journal.ppat.1004895

**Published:** 2015-05-12

**Authors:** Leena-Maija Vanha-aho, Ines Anderl, Laura Vesala, Dan Hultmark, Susanna Valanne, Mika Rämet

**Affiliations:** 1 Laboratory of Experimental Immunology, BioMediTech, University of Tampere, Tampere, Finland; 2 Laboratory of Genetic Immunology, BioMediTech, University of Tampere, Tampere, Finland; 3 Department of Molecular Biology, Umeå University, Umeå, Sweden; 4 Department of Pediatrics, Tampere University Hospital, Tampere, Finland; 5 PEDEGO Research Center, and Medical Research Center Oulu, University of Oulu and Department of Children and Adolescents, Oulu University Hospital, Oulu, Finland; Stanford University, UNITED STATES

## Abstract

The cellular immune response against parasitoid wasps in *Drosophila* involves the activation, mobilization, proliferation and differentiation of different blood cell types. Here, we have assessed the role of Edin (elevated during infection) in the immune response against the parasitoid wasp *Leptopilina boulardi* in *Drosophila melanogaster* larvae. The expression of *edin* was induced within hours after a wasp infection in larval fat bodies. Using tissue-specific RNAi, we show that Edin is an important determinant of the encapsulation response. Although *edin* expression in the fat body was required for the larvae to mount a normal encapsulation response, it was dispensable in hemocytes. *Edin* expression in the fat body was not required for lamellocyte differentiation, but it was needed for the increase in plasmatocyte numbers and for the release of sessile hemocytes into the hemolymph. We conclude that *edin* expression in the fat body affects the outcome of a wasp infection by regulating the increase of plasmatocyte numbers and the mobilization of sessile hemocytes in *Drosophila* larvae.

## Author Summary

The events leading to a successful encapsulation of parasitoid wasp eggs in the larvae of the fruit fly *Drosophila melanogaster* are insufficiently understood. The formation of a capsule seals off the wasp egg, and this process is often functionally compared to the formation of granulomas in vertebrates. Like granuloma formation in humans, the encapsulation process in fruit flies requires the activation, mobilization, proliferation and differentiation of different blood cell types. Here, we have studied the role of Edin (elevated during infection) in the immune defense against the parasitoid wasp *Leptopilina boulardi* in *Drosophila* larvae. We demonstrate that *edin* expression in the fat body (an immune-responsive organ in *Drosophila* functionally resembling the mammalian liver) is required for a normal defense against wasp eggs. Edin is required for the release of blood cells from larval tissues and for the subsequent increase in circulating blood cell numbers. Our results provide new knowledge of how the encapsulation process is regulated in *Drosophila*, and how blood cells are activated upon wasp parasitism. Understanding of the encapsulation process in invertebrates may eventually lead to a better knowledge of the pathophysiology of granuloma formation in human diseases, such as tuberculosis.

## Introduction

Parasitoid wasps are natural enemies of insects such as the fruit fly *Drosophila melanogaster*. In the course of a successful wasp infection, a female wasp lays an egg in a fruit fly larva and the wasp larva hatches. Thereafter, the wasp larva develops inside the *Drosophila* larva using the host tissue as a source of nutrition to ultimately emerge as an adult wasp, unless the wasp larva is eliminated by the host’s immune response [1].

The initial oviposition of a wasp egg triggers changes in gene expression in the fruit fly and activates both humoral and cellular defense mechanisms [2–4]. The role of the humoral defense, i.e. the production of antimicrobial peptides by the fat body, via the Imd and Toll pathways in response to a microbial challenge, is well characterized in response to microbial challenge (reviewed in [5, 6]). However, in the context of wasp parasitism, cellular immunity is more striking than the humoral response. The cellular immune responses are mediated by three types of blood cells, or hemocytes: plasmatocytes, lamellocytes and crystal cells (reviewed for example in [7, 8]). The round and small plasmatocytes are the most abundant type tallying up to 95% of all of the larval hemocytes. Plasmatocytes are responsible for phagocytosing invading microorganisms and apoptotic particles and are also required for a normal resistance against bacteria [9–12]. Crystal cells comprise around 5% of all hemocytes and they contain phenoloxidase-containing crystals that are released in the melanization response [13]. Lamellocytes, on the other hand, are solely found in larvae and are rarely present in individuals that are not immune-challenged. The main task of lamellocytes is to participate in encapsulating objects that are too large to be phagocytosed, such as the eggs of parasitoids wasps. However, the encapsulation of wasp eggs requires the concerted action of all three types of hemocytes [7].

Upon a wasp infection, the presence of a wasp egg is first recognized. Plasmatocytes are the first cells that adhere to the wasp egg and they spread around the surface of the egg forming the first layer of the capsule [14]. A wasp infection also leads to the differentiation of a large number of lamellocytes [15–17], which migrate towards the wasp egg and attach onto the plasmatocyte-covered egg. During a successful immune response lamellocytes, together with plasmatocytes, form a multilayered capsule that surrounds the wasp egg. The capsule is melanized, phenol oxidases and reactive oxygen species are released within the capsule [18], and the wasp is ultimately killed.

Although many pathways, such as the Toll and JAK/STAT pathway, have been shown to have a role in the encapsulation response [3], the phenomenon is still insufficiently understood. In this current study, we investigate the role of Edin (elevated during infection) in a wasp infection. Edin is a small peptide that is secreted into the hemolymph upon infection [19, 20], and it is required for the immune response against *Listeria monocytogenes* [21]. Earlier, we have shown that the expression of *edin* is induced after a bacterial infection, and it has a minor role in the resistance against *Enterococcus faecalis* [20]. In this study, we investigated whether *edin* expression is induced by a wasp infection using the *Leptopilina boulardi* strain G486. We also examined the role of Edin in the encapsulation response and in the activation and formation of hemocytes upon a wasp infection. We report that *edin* expression is required in the fat body upon a wasp infection in order to mount an effective encapsulation response, and that knocking down *edin* in the fat body causes defects in hemocyte mobilization in *Drosophila* larvae.

## Results

### *Edin* is induced upon a wasp infection

**Fig 1:**
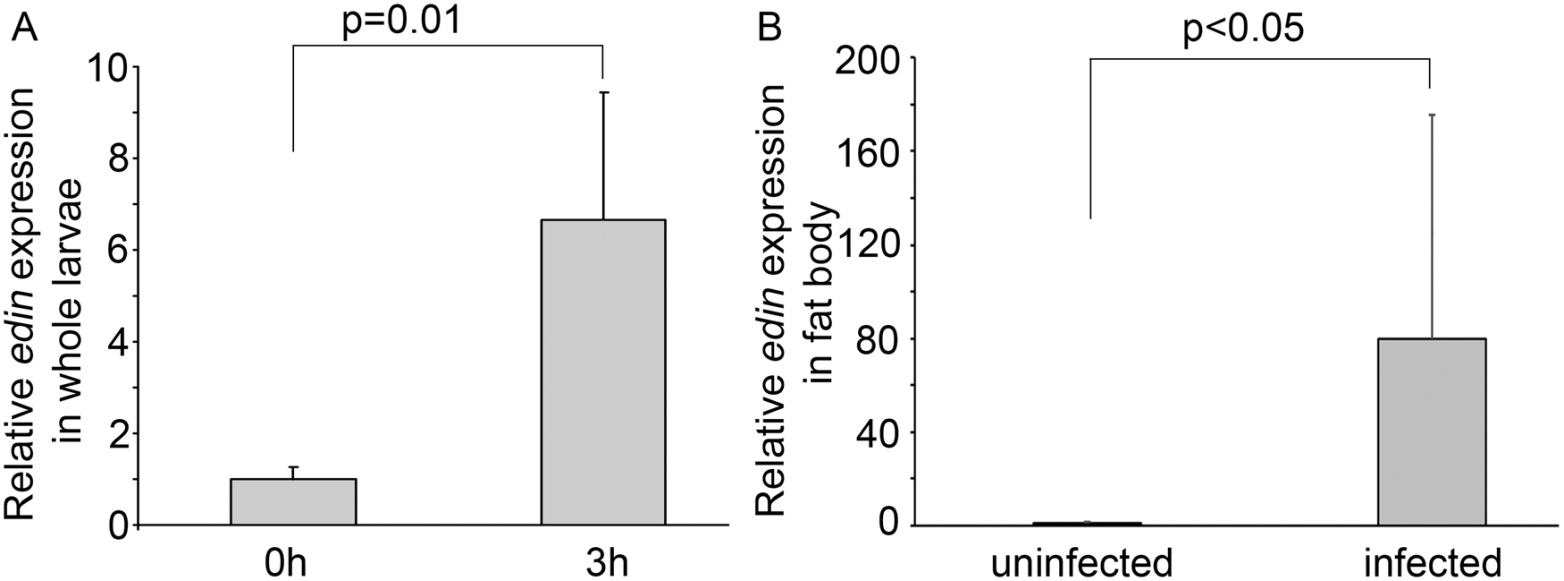
*Edin* expression is induced upon a wasp infection. **(A)** Wasp infection causes a 6.7-fold increase in *edin* expression in 2^nd^ instar *Canton S* larvae. Data are pooled from two independent experiments, n = 2 for each experiment, where one sample represents 10 larvae. **(B)** *Edin* expression is induced in the fat bodies of *Canton S* larvae 24 hours post infection. The data are pooled from four independent experiments, and each experiment consisted of two samples, where one sample represents 8–10 larval fat bodies.

We have previously shown that *edin* is induced both *in vitro* and *in vivo* upon a microbial infection, but were unable to find any essential role for Edin in this context [20]. To test whether a wasp infection induces the expression of *edin*, we infected *Canton S* larvae with the parasitoid wasp *Leptopilina boulardi* strain G486, and determined the expression levels of *edin* in whole larvae three hours after infection using qRT-PCR. As is seen in Fig 1A, the wasp infection led to a 7-fold induction in the expression levels of *edin* compared to uninfected larvae. Because the fat body is the main immune-responsive organ in the fruit fly, we next looked at *edin* mRNA levels in the fat bodies of wasp-infected larvae 24 hours post-infection. As is shown in Fig 1B, the expression of *edin* was more highly induced in the fat bodies of the wasp-infected larvae than in whole larvae (80-fold induction). Our results indicate that *edin* is upregulated after a wasp infection in larvae and that the fat body is a main source for its expression.

### *Edin* expression in the fat body is required for the normal encapsulation of wasp eggs

**Fig 2:**
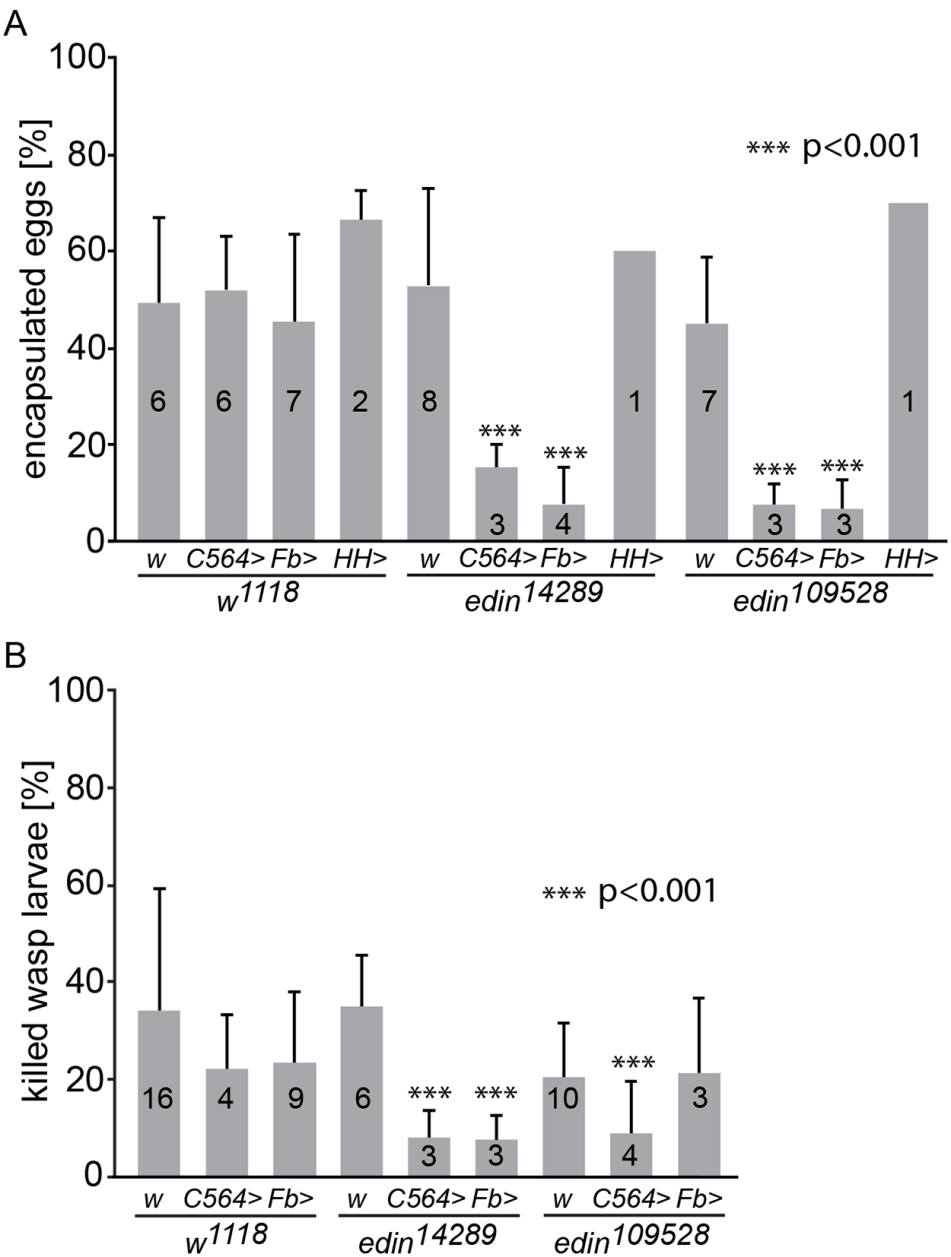
Knock down of *edin* in the fat body decreases the encapsulation and killing ability of *Drosophila* larvae. **(A)** The encapsulation response of two different *edin* RNAi lines (*edin* ^*14289*^ and *edin* ^*109528*^) was analyzed 27-29h after a wasp infection. The *C564*-*GAL4* (*C564>)*, *Fb-GAL4* (*Fb>*) and *Hml* ^*Δ*^;*He-GAL4* (*HH>*) drivers were used to drive the expression of the RNAi constructs. *w* ^*1118*^ (*w*) was used as control. Data were pooled from one to eight individual experiments, as depicted on each column, each experiment with at least 50 analyzed individual infected larvae. **(B)** The ability of *Drosophila* larvae to kill wasp eggs was assessed with two different *edin* RNAi lines (*edin* ^*14289*^ and *edin* ^*109528*^) 48-50h after infection. The *C564*-*GAL4* (*C564>*) and *Fb-GAL4* (*Fb>*) drivers were used to drive the expression of the RNAi constructs. *w* ^*1118*^ (*w*) was used as control. Data are pooled from three to sixteen independent experiments, as indicated on each column, and at least 50 infected larvae were scored per experiment. Error bars in A and B show standard deviations. Knocking down the expression of *edin* in several tissues including the fat body or in the fat body alone caused a significant decrease in the encapsulation activity and killing response of *Drosophila* larvae compared to controls, whereas knocking down *edin* in hemocytes had no effect.

Fruit fly larvae can mount an effective immune response against invading parasitoids by encapsulating the wasp egg. To address the functional significance of *edin* expression for the encapsulation process upon an *L*. *boulardi* infection, we used the UAS-GAL4 system to knock down *edin* expression. The normal response against the wasp egg is the formation of a visible melanized capsule around the parasitoid egg, and in our hands, 45–66% of control larvae had a melanized capsule. First, we crossed *edin* ^*14289*^ RNAi flies (#14289, hereafter referred to as *edin* ^*14289*^) with flies carrying the *C564-GAL4* driver, which is expressed in many organs, including the fat body, salivary glands and lymph glands [22], and looked for the presence of melanized capsules 27–29 hours after the wasp parasitization (Fig 2A). Parasitized *w* ^*1118*^ controls showed an encapsulation rate of 47%. Similarly, *w* ^*1118*^ crossed with *C564-GAL4* or *edin* ^*14289*^ showed encapsulation rates of 52% and 53%, respectively, while only 15% of *edin* ^*14289*^ crossed with *C564-GAL4* showed melanized capsules. To ensure that the observed phenotype was caused by reduced *edin* expression, we analyzed the encapsulation response of another *edin* RNAi line (#109528, hereafter referred to as *edin* ^*109528*^). Similarly to the *edin* ^*14289*^ line, *edin* ^*109528*^ crossed with the driver line showed a clearly decreased encapsulation efficiency of 7% (Fig 2A), when compared to *edin* ^*109528*^ crossed with *w* ^*1118*^.

We next used a fat body-specific driver to examine specifically whether the lowered encapsulation response was due to the role of *edin* in the fat body. We crossed both the *edin* ^*14289*^ and *edin* ^*109528*^ RNAi lines with the *Fb-GAL4* driver line and examined the encapsulation response of the offspring. *Fb-GAL4* crossed with *w* ^*1118*^ showed encapsulation levels of 45% (Fig 2A), whereas *edin RNAi* flies crossed with *Fb-GAL4* showed an encapsulation activity of only 8% (*edin* ^*14289*^) and 7% (*edin* ^*109528*^). In addition, similar results were also obtained with another fat body-specific driver, *Lsp2-GAL4* (*edin* ^*109528*^, S1 Fig).

We also analyzed the encapsulation activity of *edin* RNAi larvae crossed with the pan-hemocyte driver *Hml* ^*Δ*^;*He-GAL4* and were not able to see any effect with either of the RNAi lines (60% and 70% encapsulation, Fig 2A). Together, these data suggest that Edin is required for a normal encapsulation response after parasitization, and that its expression is required in the larval fat body but not in the hemocytes.

### *Edin* expression is required for the resistance against wasp parasitism in *Drosophila* larvae

Scoring for the ability of the fly larva to melanize the wasp egg does not indicate whether the fruit fly larva is actually able to overcome the parasitization. Therefore, we replicated the experimental setting in Fig 2A, but scored for the presence of living or dead wasp larvae 48–50 hours post infection. The parasite was scored as killed by the fruit fly larva if a melanized wasp egg was found in the hemocoel in the absence of a living wasp larva. As is seen in Fig 2B, the percentage of dead wasps in control larvae varied between 20–34%. When *edin* ^*14289*^ RNAi was induced with either the *C564-GAL4* or *Fb-Gal4* driver, the percentage of dead wasps was significantly reduced (8% in both cases). A significant decrease was also observed with the combination of the *edin* ^*109528*^ RNAi line and the *C564-GAL4* driver (9% killing rate). These results, together with the encapsulation phenotype, indicate that *edin* is required for the resistance against wasp parasitism in *Drosophila* larvae.

### *Edin* expression is not required for lamellocyte differentiation in *Drosophila* larvae upon *L*. *boulardi* parasitism

**Fig 3:**
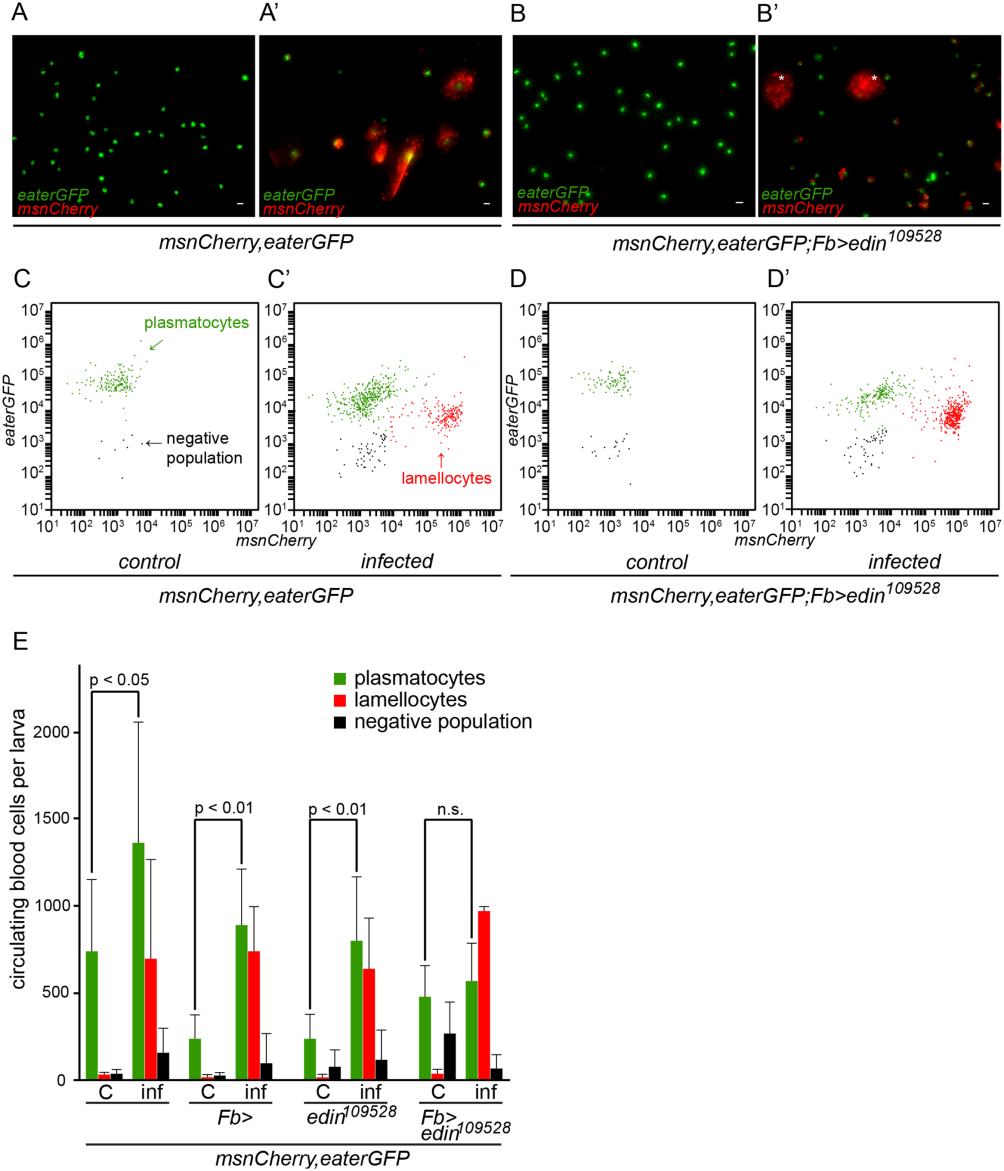
Quantification of hemocytes in *edin* RNAi larvae after a wasp infection. **(A-B)** Hemocytes of infected larvae were bled 48–50 hours post-infection and visualized with the *eaterGFP* (green) and *msnCherry* (red) reporters. Uninfected controls contained only GFP-positive cells that corresponded to plasmatocytes (green). (**A’ and B’**) *msnCherry* expression was detected in the infected samples and this included lamellocytes (asterisks) and cells that express both *eaterGFP* and *msnCherry* indicating that they were undergoing lamellocyte transition. Lamellocytes were present also in the infected *edin* RNAi larvae suggesting that *edin* expression is not necessary for lamellocyte differentiation. Scale bars are 10 μm **(C-E)** Flow cytometry was carried out to quantify the amount of hemocytes in the unchallenged and the wasp infected *edin* RNAi larvae. (C = control, inf = infected)

Lamellocytes have a central role in the resistance against *L*. *boulardi* parasitism. They are not found in the hemocoel of healthy, unchallenged *Drosophila* larvae, but they are formed in response to a wasp infection [15–17]. To investigate whether the expression of *edin* in the fat body is required for lamellocyte formation, we bled hemocytes of wasp-challenged larvae 48–50 hours after infection. Plasmatocytes and lamellocytes were visualized using the *eaterGFP* (green) and *msnCherry* (red) reporters, respectively. As is shown in Fig 3A and 3B, all of the hemocytes in the unchallenged larvae express the *eaterGFP* reporter and are *msnCherry*-negative, indicating that only plasmatocytes are present. Lamellocytes are *msnCherry*-positive, large, and flat cells. They are present only in the infected larvae (Fig 3A’ and 3B’) and are found both in RNAi treated and control larvae, indicating that *edin* expression in the fat body is not required for lamellocyte formation upon a wasp infection (Fig 3B’). It is noteworthy that the infected larvae contain cells that express both *eaterGFP* and *msnCherry* reporters, showing that some of the cells are undergoing plasmatocyte to lamellocyte transition and are not yet fully differentiated lamellocytes (Fig 3A’ and S2 Fig).

In order to obtain additional information about the role of Edin after wasp infection, we used flow cytometry and the *msnCherry*,*eaterGFP* reporter to analyze hemocytes of larvae, where *edin* was knocked down in the fat body. Fig 3C–3D’ show representative scatter plots of hemocytes of uninfected and infected larvae with *edin* RNAi in the fat body as well as age-matched uninfected and infected control larvae at the 27–29 hour time point. Lamellocytes were induced in spite of *edin* depletion in the fat body. When comparing hemocyte numbers of uninfected and infected control larvae and *edin* RNAi larvae, we found that although lamellocyte numbers of infected animals did not differ (p = 0.061, Fig 3E), the plasmatocyte numbers generally increased approximately two to three fold after infection in controls but remained constant in *edin* knock-down larvae (Fig 3E). Taken together, Edin was dispensable for lamellocyte formation but seemed to be necessary to increase plasmatocyte numbers after a wasp infection.

### Edin expression in fat body is not necessary for plasmatocyte spreading and adhesion

**Fig 4:**
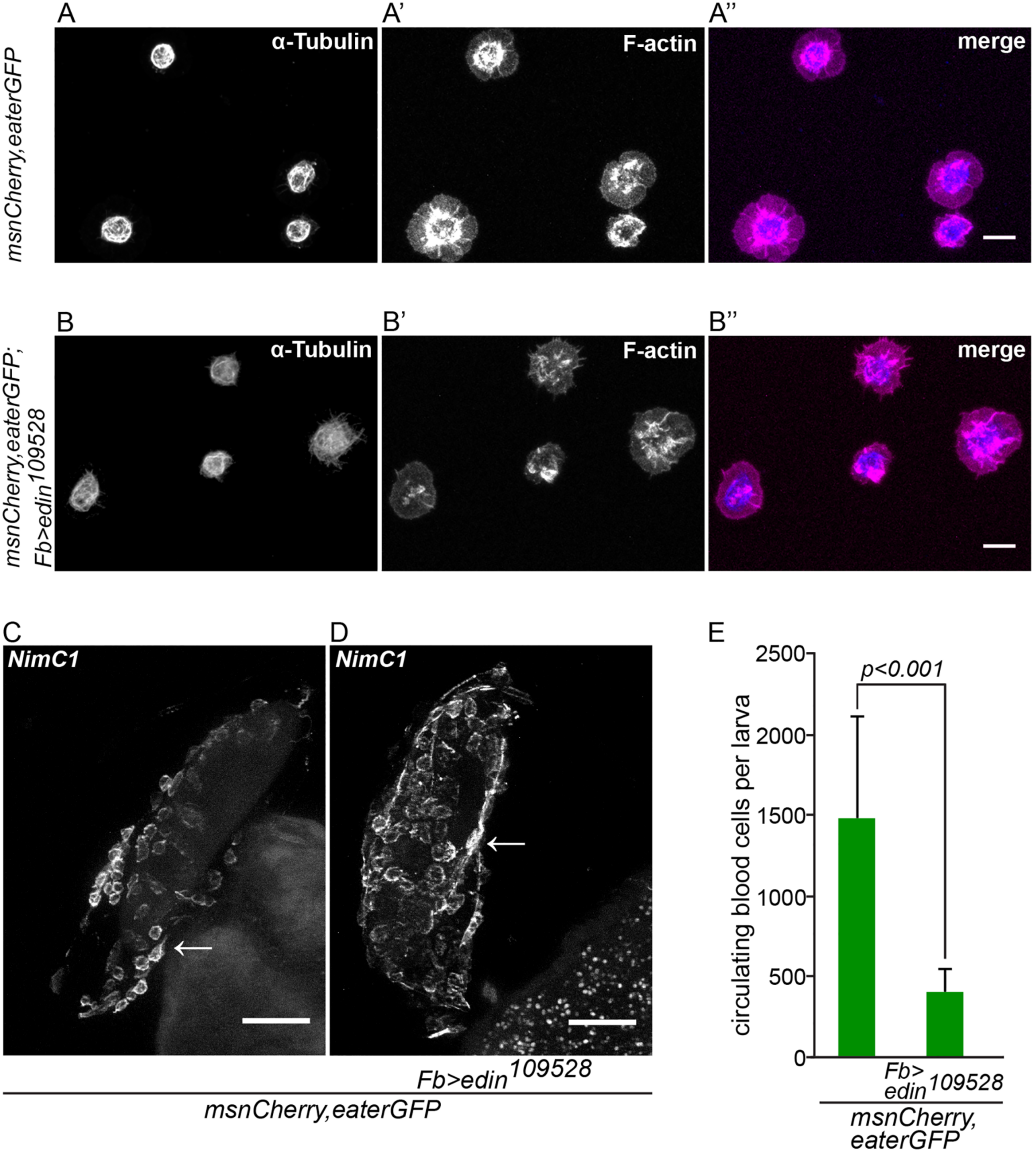
*Edin* expression in fat body is dispensable for normal hemocyte attachment to and spreading on glass and wasp eggs, but is necessary to increase blood cell numbers in circulation early after wasp infection. (**A-B”**) Hemocytes from infected control larvae (*msnCherry*,*eaterGFP*, A-A”) and from infected larvae in which *edin* was knocked down in the fat body (*msnCherry*,*eaterGFP;Fb>edin* ^*109528*^, B-B”) spread normally on glass 14 hours after wasp infection despite knock down of *edin* in fat body. The spreading ability of hemocytes was assayed by staining α-Tubulin (blue) and F-actin (magenta). The size bar denotes 10 μm. (**C and D**). Wasp eggs from infected control larvae (*msnCherry*,*eaterGFP*, C) and from infected larvae in which *edin* was knocked down in the fat body (*msnCherry*,*eaterGFP;Fb>edin* ^*109528*^, D) were stained with the anti-plasmatocyte antibody *NimC1*. The wasp eggs were dissected 14 hours after parasitization and are still attached to the gut. Plasmatocytes spread normally on the eggs irrespective of *edin* RNAi in the fat body. Arrows denote examples of plasmatocytes spreading and adhering normally on the surface of the wasp egg. The scale bar depicts 50 μm. (**E**) *Edin* RNAi in the fat body (*msnCherry*,*eaterGFP;Fb>edin* ^*109528*^) reduced the number of circulating cells after wasp infection in comparison to control larvae (*msnCherry*,*eaterGFP*) 14 hours after infection. Circulating blood cell numbers were obtained with flow cytometry.

In order to properly encapsulate wasp eggs, blood cells must adhere and spread on the egg surface until the egg is finally encapsulated. The Rac GTPase *Rac2* regulates the actin cytoskeleton that mediates the spreading of plasmatocytes on the wasp egg [23]. To ensure that the defect in encapsulation is not caused by a defective plasmatocyte function, we tested whether plasmatocytes adhere and spread normally on glass slides and on wasp eggs. In our experimental setting, lamellocytes appear 20 hours after parasitization. To get only plasmatocytes, we bled larvae 14 hours after wasp infection and stained the microtubules and the actin cytoskeleton (Fig 4A and 4B”). We measured the tubulin to actin ratio from approximately 120 hemocytes of larvae with *edin* RNAi in fat body and control larvae, and found no significant difference in the spreading behavior (control: tubulin/actin = 0.46, standard deviation = 0.18; *edin* RNAi: tubulin/actin = 0.42, standard deviation = 0.21; p = n.s., S1 Table). Another way of looking at spreading behavior is assaying the distribution of the NimC1 protein that is specific for plasmatocytes. The NimC1 protein forms a cytoplasmic ring in control cells, whereas it accumulates in the center of the cell in *Rac2* mutants [23]. NimC1 antibody staining of plasmatocytes on the wasp egg 14 hours after parasitization of *edin* RNAi larvae was indistinguishable from controls (Fig 4C and 4D) indicating normal adhesion and spreading of plasmatocytes *in vivo*.

### Edin expression in the fat body is required for the increase of plasmatocyte numbers in circulation after a wasp infection

The defining early events of capsule formation are the recognition of the wasp egg by plasmatocytes [14] and a significant increase of hemocytes in circulation. [24]. To study whether *edin* expression is required to increase plasmatocyte numbers in the early stages of an infection, we counted plasmatocytes 14 hours after wasp infection using flow cytometry. As is shown in Fig 4E, *edin* RNAi in the fat body resulted in more than three times fewer cells compared to controls (p<0.001). Taken together, Edin is dispensable for lamellocyte formation but it is necessary to increase plasmatocyte numbers in circulation in the early stages of a wasp infection.

### Knocking down *edin* in the fat body causes an altered hemocyte phenotype in wasp-infected larvae

**Fig 5:**
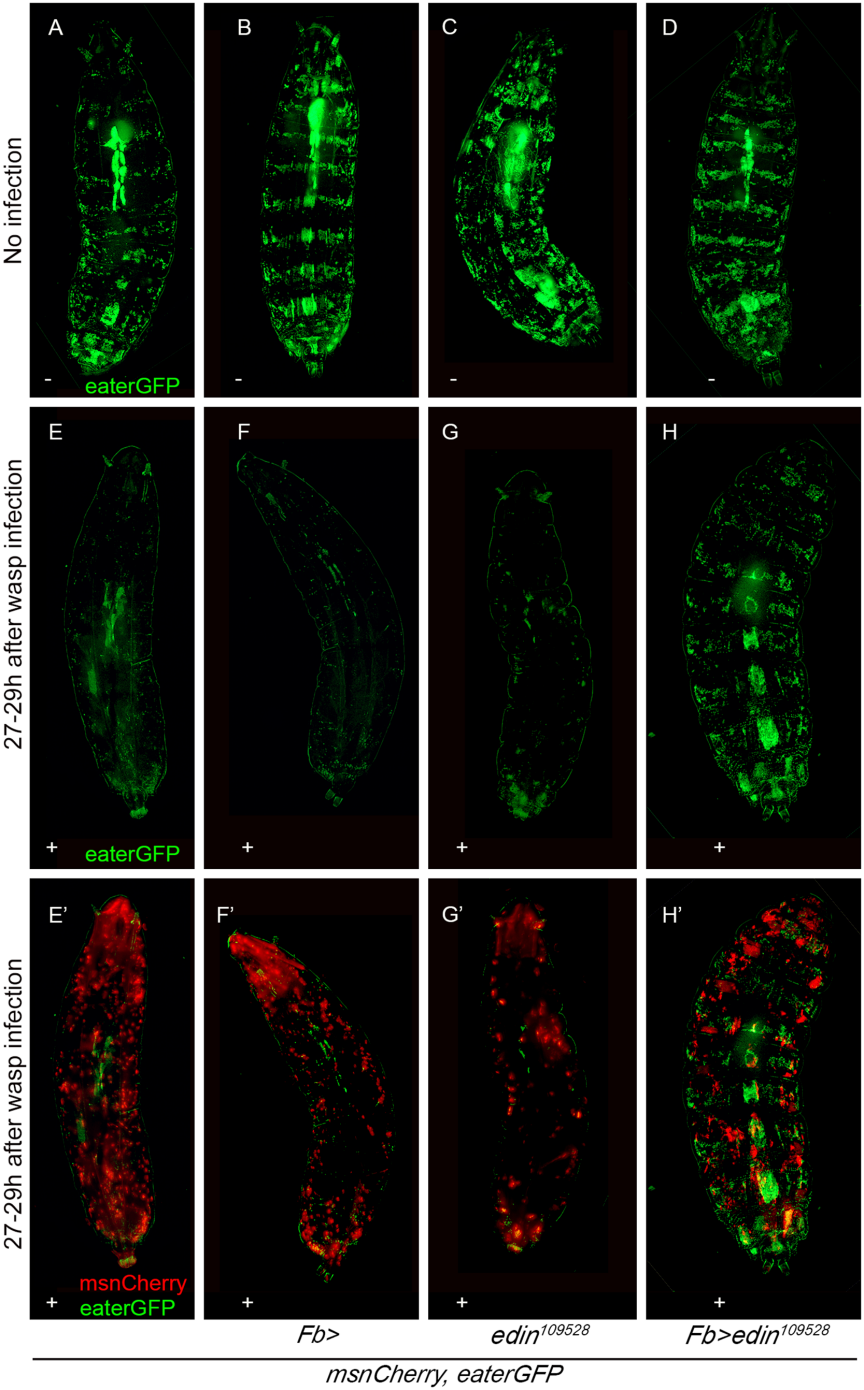
*Edin* expression in the fat body is required for the activation of plasmatocytes upon a wasp attack 27–29 hours after infection. The *in vivo* phenotype of wasp infected *edin* RNAi larvae was studied using the *eaterGFP* (green = plasmatocytes) and *mCherry* (red = lamellocytes) reporters. Imaging was performed 27–29 hours post infection with living *Drosophila* larvae. **(A-D)** Uninfected larvae show an uninterrupted banding pattern formed by sessile plasmatocytes (green). **(E-H)** Shows only the green channel (eaterGFP) of infected larvae and **(E’-H’)** both the green and the red (msnCherry) channel. Infected larvae have lost the banding pattern and lamellocytes have appeared, but infected *msnCherry*,*eaterGFP;Fb>edin* ^*109528*^ larvae still show a visible banding pattern formed by the sessile cells.*–* = uninfected larvae, + = wasp infected larvae. Fig 5 shows representative images of at least 10 larvae per condition and per genotype.

Sessile plasmatocytes reside attached to the skin of *Drosophila* larvae and form a hematopoietic compartment that releases blood cells in response to a wasp infection [25, 26]. In order to see if the decreased numbers of plasmatocytes were due to a defect in releasing the sessile plasmatocytes into circulation, we imaged the *Fb-GAL4-*driven *edin* RNAi larvae and the respective control crosses 27–29 hours after the wasp parasitization, and again used the *msnCherry*,*eaterGFP* reporter line to allow the visualization of plasmatocytes (green) and lamellocytes (red). In the uninfected controls (Fig 5A–5D, top row), the banded pattern of plasmatocytes and the lymph gland could been seen. The bands represented plasmatocytes that resided in the sessile compartment in the absence of an immune stimulus. When the larvae were infected by wasps, the green banded pattern disappeared (Fig 5E–5G) and lamellocytes appeared in the hemolymph (Fig 5E’–5G’). This was due to the activation of the hemocytes in the sessile compartment in response to the wasp infection, which causes the cells to leave the compartment and enter the circulation, where many differentiate into lamellocytes [25, 26]. Consistent with our flow cytometry data (Fig 3), when *edin* was knocked down in the fat body, lamellocytes still appeared in the circulation showing that Edin did not affect the formation of lamellocytes (Fig 5H’). However, in the *edin* knockdown larvae the banded pattern of plasmatocytes was not disrupted as in the controls (Fig 5H and 5H’). Of note, overexpression of *edin* in the fat body did not disrupt the banded pattern indicating that the overexpression of *edin* alone was not sufficient for releasing the sessile hemocytes into the circulation (S3 Fig). In conclusion, our data suggest that *edin* expression in the fat body affects plasmatocyte activation and release from the sessile compartment. This suggests that the silencing of *edin* results in a compromised response to *L*. *boulardi* parasitism in the early stages of the infection, and that the altered resistance is due to insufficient plasmatocyte numbers in circulation.

## Discussion

Encapsulation is a complex response against a wasp attack in fruit fly larvae and it requires the concerted action of activated hemocytes. In the course of the encapsulation response, plasmatocytes and the encapsulation-specific lamellocytes form a multilayered capsule around the wasp egg and sequester the invading parasite from the hemocoel of the larva. In addition to inducing the encapsulation response, a wasp infection causes changes in the expression profile of the fruit fly genes [3, 4]. Our results show that *edin* was rapidly induced in response to an infection by the endoparasitoid wasp *Leptopilina boulardi* and that *edin* expression in the fat body, but not in hemocytes, was required to mount a normal encapsulation response against the wasp. Encapsulation was not blocked entirely, however, as approximately 10% of the larvae encapsulated the wasp egg, when *edin* was knocked down in the fat body. Nevertheless, lamellocyte numbers were unaffected and plasmatocyte spreading behavior was normal. Instead, in larvae where *edin* was knocked down in the fat body, fewer plasmatocytes were present in circulation, while more hemocytes were retained within the sessile compartment. These data indicate that the presence of lamellocytes alone is not enough for the fruit fly larva to kill the wasp egg. Sufficient numbers of plasmatocytes are also needed.

We discovered that knocking down *edin* in the fat body did not affect lamellocyte differentiation but compromised the increase of plasmatocyte numbers after a wasp infection. The impaired encapsulation response observed in our study could be therefore due to the misregulation of hemocyte proliferation and/or activation. Because plasmatocyte function was not impaired, as the cells were able to attach and spread normally onto glass slides and wasp eggs, the lowered plasmatocyte number could be the cause of the defects observed in the encapsulation response. Other studies have shown that high hemocyte numbers are associated with an increased resistance against parasitoid wasps in *D*. *melanogaster* as well as in other *Drosophila* species [27–30], although the molecular mechanisms behind this phenomenon are not understood. In our study, the lowered numbers of plasmatocytes are observed already early on during the wasp infection (14 h post infection), suggesting that the function of Edin is critical at the onset of an immune response. This might be the case also in the context of an antimicrobial response, where *edin* knock down seems to have a modest effect on the levels of some antimicrobial peptides during the early phases of a bacterial infection [20].

Studies have shown that, when hemocytes are activated after an immune stimulus, the banded pattern formed by plasmatocytes is disrupted and the cells are released into the circulation [25, 26, 31], where they can differentiate into lamellocytes [16, 17, 26]. The mobilization of sessile cells occurs prior to the release of hemocytes from the lymph gland [17, 26], and this disruption of the banded pattern is caused by changes in the adhesive properties of the cells. Several genes have been reported to be involved in the attachment of the sessile hemocytes to the sessile compartment [25, 32]. For example, the conserved Rho family of GTPases, namely Rac1 and Rho, regulate the release of sessile cells through the regulation of the adhesive properties of the cells [33, 34]. It has also been suggested that sessile hemocytes adhere to laminin under the larval integument in a syndecan-dependent manner [35]. Additionally, the EGF-repeat containing receptor Eater, which was originally identified for its role in the phagocytosis of bacteria [36], was recently reported to be required in plasmatocytes for the adhesion of hemocytes to the sessile compartment [37]. In our current study, we show that sessile plasmatocytes of *edin* RNAi larvae did not leave the sessile bands, and the numbers of circulating plasmatocytes did not change after a wasp infection, yet normal amounts of lamellocytes were formed. Despite comparatively normal amounts of lamellocytes, the encapsulation response was impaired when the sessile plasmatocytes could not be mobilized. Hence, besides forming the first layer of the capsule and giving rise to lamellocytes, plasmatocytes have other functions in the encapsulation response that are dependent on *edin* expression in the fat body.

**Fig 6:**
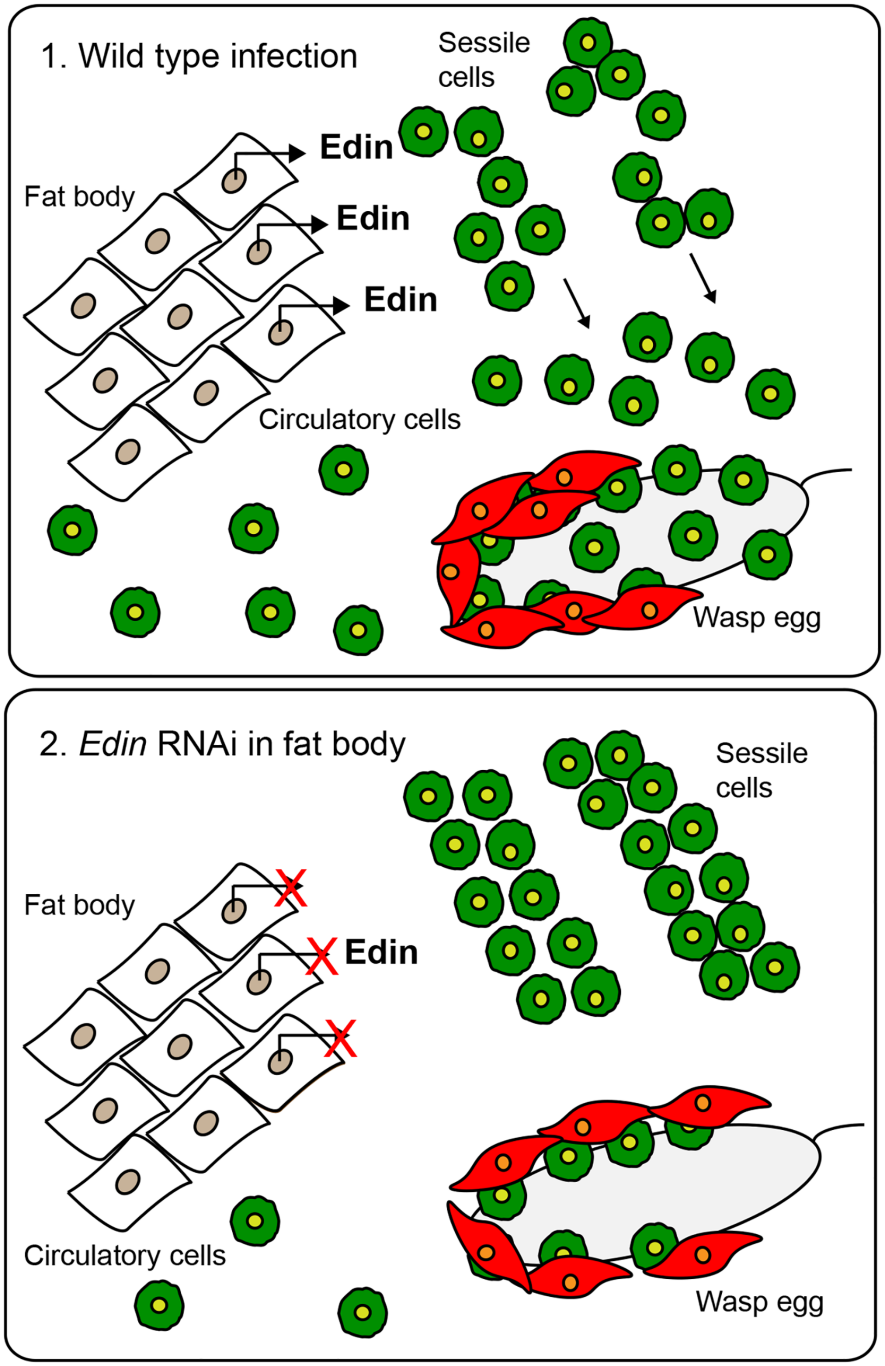
A schematic presentation of the function of Edin. (1.) *Edin* is induced in the fat body shortly after wasp infection and secreted into the hemolymph. There, Edin directly or indirectly induces the release of plasmatocytes from the sessile hemocyte compartment. These cells go into circulation, find the wasp egg and participate in forming the capsule around the parasitoid egg. (2.) If the expression of *edin* is knocked down in the fat body in the context of a wasp infection, plasmatocytes are retained in the sessile compartment instead of being released into circulation, causing a defect in the encapsulation of the wasp egg.

Our results imply that the effect of Edin is non-cell autonomous and that it seems to act as a molecule that signals from fat body to hemocytes either directly or indirectly. Although the humoral and cellular aspects of *Drosophila* immunity are often depicted as separate, several studies have provided evidence of the interaction between hemocytes and the fat body. For example, the antimicrobial peptide response to an *E*. *coli* infection in *domino* mutants which lack hemocytes, is normal, but these mutants fail to induce *Diptericin* during a gut infection by *Erwinia carotovora* suggesting that hemocytes mediate a signal from the gut to the fat body [38, 39]. In line with these data, Brennan et al. have shown that Psidin acts in the hemocytes to activate the production of Defensin in the fat body [40]. Another example of crosstalk between hemocytes and the fat body is the requirement of *Upd3* expression in hemocytes to activate the JAK-STAT pathway in the fat body of adult flies [41]. Furthermore, in larvae, the production of the cytokine Spätzle by hemocytes is needed for the activation of Toll-mediated AMP production in the fat body [42]. Hemocytes are also mediators of the transport of the nitric oxide from its site of production in the gut epithelia to the fat body, where AMP production via the Imd pathway is activated [43, 44]. However, contradicting data also exist for adult flies showing that the ablation of hemocytes by apoptosis does not affect AMP induction in the fat body [45, 46]. A more recent study has shown that the interaction between the fat body and hemocytes is crucial in controlling tumor cell death [47]. Recently, we also showed that Toll signaling in the fat body controlled hemocyte differentiation and activation, but that it did not play a major role in the immune response against *L*. *boulardi* as the wasps were able to suppress Toll signaling in the fat body [48]. These examples point to the existence of active tissue-to-tissue signaling that orchestrates appropriate immune responses against different immune challenges. According to our results, Edin functions as a cytokine-like molecule, but the receptor for Edin and its localization remain to be studied. Edin might signal directly from the fat body to the hemocytes, but it may also signal to other tissues or cells that then affect the function of the hemocytes in the sessile compartment (Fig 6). Although Edin is not structurally conserved outside brachyrecan flies [20], its cytokine-like function might be conserved, as in the case of the Spätzle-like function of the vertebrate nerve growth factor β [49], for example.

Based on our results Edin appears to be a key regulator in the cross-talk between fat body and hemocytes in the context of a wasp infection. As in the encapsulation response, the granuloma formation in vertebrates also requires the recruitment of many different cell types. For example, the adult zebrafish responds to a *Mycobacterium marinum* infection by enclosing the infectious foci in granulomas [50, 51], but also the intracellular bacterium *Listeria monocytogenes* is sequestered inside granulomas to constrain the infection [52]. Whether information obtained from genetically tractable model organisms such as *Drosophila melanogaster*, will lead to a better understanding of the pathophysiology of granuloma formation remains to be studied.

## Materials and Methods

### *Drosophila* stocks

*UAS-edin* RNAi (CG32185) flies #109528 and #14289 (hereafter called *edin* ^*109528*^ and *edin* ^*14289*^) were obtained from the Vienna Drosophila Resource Center. The driver lines used in this study were the fat body-specific driver *Fb-GAL4*, the hemocyte-specific driver *Hml* ^*Δ*^;*He-GAL4* [48] and *C564-GAL4*, which was obtained from Prof. Bruno Lemaitre (Global Health Institute, EPFL, Switzerland). The *C564-GAL4* driver is expressed in many tissues such as the fat body, lymph gland, salivary glands, gut and brain but not in hemocytes [22].

The hemocyte reporter lines *eaterGFP* (for plasmatocytes) [53] and *MSNF9mo-mCherry* (for lamellocytes, hereafter called *msnCherry*) [54] were obtained from Robert Schulz’s laboratory. The lines were crossed to create the *msnCherry*,*eaterGFP* reporter line. The *mCherry*,*eaterGFP* reporter was further crossed with *Fb-GAL4* and *edin RNAi* ^*109528*^ to obtain the *mCherry*,*eaterGFP;Fb-GAL4* and *mCherry*,*eaterGFP;edin* ^*109528*^ lines. *Canton S* flies were used for RNA extractions.

### Wasp infection

Ten *GAL4-*driver virgin females were crossed with five RNAi male flies and allowed to lay eggs at +25°C. *w* ^*1118*^ flies and *GAL4-*driver virgin females crossed with *w* ^*1118*^ males and *w* ^*1118*^ virgin females crossed with RNAi males were used as controls. The flies were transferred daily into fresh vials and the vials containing eggs were transferred to +29°C. On the third day after egg-laying, the larvae were infected with 20 female and 10 male wasps of the *Leptopilina boulardi* strain G486. The larvae were infected for 2 hours at room temperature after which the wasps were removed and the larvae were transferred back to +29°C.

The encapsulation properties were assayed 27–29 hours after the infection and the killing ability of the larval immune system 48–50 hours after the wasp infection. The egg was scored as encapsulated when traces of melanin were found on it. To analyze the killing ability of the *Drosophila* larva, three types of phenotypes were scored. The wasp was scored as killed if a melanized wasp egg or melanized wasp larva without other living wasp larvae was found in the hemolymph, whereas the wasp was scored as living when a living wasp that had escaped a melanized capsule was present or when a living wasp larva without any melanized particles was found in the hemocoel.

### RNAi extraction from larvae and fat bodies

Eight to ten *Canton S* larvae per sample were snap frozen on dry ice at 0 hours or 3 hours after the wasp infection. The fat bodies were dissected in 1x PBS 24 hours after the wasp infection and kept on ice. Both larvae and fat bodies were homogenized in TRIsure reagent (Bioline, London, UK) and total RNAs were extracted according to the manufacturer’s instructions.

### Quantitative real-time PCR

Quantitative RT-PCR was carried out using the iScript One-Step RT-PCR kit with SYBR Green (Bio-Rad, Hercules, CA, USA) and the Bio-Rad CFX96 (Bio-Rad) instrument according to the manufacturer’s instructions. Results were analyzed with the Bio-Rad CFX Manager software version 1.6. *Actin5C* was used as a housekeeping gene. The following primers were used: Forward 5’-CTCGTGTCCTGCTGTCTG-3’ and reverse 5’-GCCTTCGTAGTTGTTCCG-3' for *edin* and forward 5’-CGAAGAAGTTGCTGCTCTGG-3’ and reverse 5’-AGAACGATACCGGTGGTACG-3’ for *Actin5C*.

### Microscopy

*Drosophila* larvae were imaged using 3^rd^ instar larvae 27–29 hours after the wasp infection. The larvae were washed three times in H_2_O and embedded on microscope slides in a drop of ice-cold glycerol. The larvae were immobilized at -20°C before imaging. The Zeiss ApoTome.2 was used for live imaging of larvae. For hemocyte imaging, the larvae were washed three times in H_2_O, and the hemocytes were bled into 1 x PBS 48–50 hours after the wasp infection. Uninfected controls of the same age were also used. The hemocytes were let to adhere to the glass surface of a microscope slide for 30 minutes, after which they were fixed with 3.7% paraformaldehyde for 5 minutes. The samples were washed with PBS and mounted with the Prolong Gold Anti-Fade reagent with DAPI (Molecular Probes). Hemocyte imaging was carried out with the Zeiss AxioImager.M2 microscope with Zeiss AxioCam and the Zen Blue 2011 software and with the Zeiss LSM780 in the case of the antibody-stained hemocytes. The hemocyte images were processed with ImageJ 1.49p (Rasband WS, ImageJ, U.S. National Institutes of Health, Bethesda, Maryland, USA, imagej.nih.gov/ij, 1997–2012).

### Quantification of larval hemocytes with flow cytometry

Hemocytes from infected and control larvae were bled into 1 x PBS with 8% BSA to obtain the hemocytes. Flow cytometry was used to detect *eaterGFP*-positive and *msnCherry*-positive cells in these samples. The Accuri C6 flow cytometer (BD, Franklin Lakes, NJ, USA) was used to run the samples, and the data was analyzed using the BD Accuri C6 software. The gating strategy is explained in S2 Fig.

### Immunofluorescence

For F-actin and α-tubulin stainings, hemocytes were bled from 15 larvae per cross into 20 μl of 1 x PBS with 8% BSA in pools of three larvae per well and allowed to spread on a glass slide for 45 minutes. Cells were fixed with 3.7% paraformaldehyde/PBS solution for 10 minutes, washed three times with PBS and permeabilized for 5 minutes with 0.1% Triton X-100 before antibody staining. Cells were incubated for 2 hours with an unconjugated mouse α-tubulin monoclonal antibody (Life Technologies, 1μg/ml concentration) followed by one hour incubation with the Alexa Fluor 405 goat anti-mouse secondary antibody (Life Technologies, a 1:500 dilution in 1% BSA in PBS). F-actin was visualized by incubating the cells for 30 minutes with the Alexa Fluor 680 nm Phalloidin stain (Invitrogen) diluted to 1:50 in 1x PBS with 1% BSA. After this, the cells were washed 3 times with PBS and mounted using the ProLong Gold antifade mountant (Life Technologies). We measured the area of Phalloidin and α-tubulin staining with ImageJ 1.49p and calculated the ratio of α-tubulin to Phalloidin areas.

Wasp eggs with hemocytes attached onto them were collected from fly larvae 12–14 hours after infection in a drop of 8% BSA in 1 x PBS, fixed with 3.7% paraformaldehyde/PBS solution for 10 minutes, washed three times with PBS, and stained for 4 hours with an undiluted mixture of monoclonal P1a and P1b (NimC1) plasmatocyte-specific antibodies [55]. Thereafter, the samples were washed 3 times with PBS and incubated with the Alexa Fluor 405 goat anti-mouse secondary antibody (Life Technologies, 1:500 dilution). The eggs were mounted with 50% glycerol prior to imaging. Three eggs per cross were imaged.

### Statistical analyses

*Edin* expression data was analyzed using an independent samples two-tailed T-test, with unequal variances assumed. The analysis was carried out using Microsoft Office Professional Plus Excel 2013. The threshold for statistical significance was established as p<0.05.

We applied a Generalized Linear Model (glm) in R 3.1.2 (2014-10-31)— “Pumpkin Helmet” (R Development Core, 2003) to analyze the encapsulation and parasite killing data (R Core Team 2014, R: A language and environment for statistical computing. R Foundation for Statistical Computing, Vienna, Austria, http://www.R-project.org/). The categorical explanatory variable was “Cross” and the binary response variable was numbers of “successful encapsulation” or “killed parasites” and numbers of “failed encapsulation” or “failed parasite killing”. Differences between specific crosses were analyzed by Chi-square tests.

We analyzed the cell spreading data and cell numbers 14–16 hours post infection with Welch’s T-test implemented in R 3.1.2 (2014-10-31) (R Development Core, 2003). The data were log-transformed prior to the analyses to obtain normal distribution.

Full factorial analysis of variance (ANOVA) was applied to data on plasmatocyte and lamellocyte numbers 27–29 hours after infection with cross and infection status (infected or not infected) as explanatory variables. The data did not meet the requirement for normal distribution and was log transformed prior to the analyses. In the analysis of plasmatocyte numbers, a significant interaction term was found between cross and infection status and therefore plasmatocyte numbers were further analyzed conducting ANOVAs separately for each cross with infection status as explanatory variable. This data was analyzed using IBM SPSS Statistics version 22.

## Supporting Information

S1 Fig Knock down of *edin* in the fat body with the *Lsp2-GAL4* driver decreases the encapsulation of *Drosophila* larvae. The encapsulation response of the *Lsp2-GAL4*-driven *edin* ^*109528*^ RNAi was analyzed 27-29h after a wasp infection. Data were pooled from two to four individual experiments, as depicted on each column, each experiment with at least 90 analyzed individual infected larvae. Statistical analyses were carried out as in Fig 2 using a Generalized Linear Model with binomial distribution. Error bars represent standard deviation.
(TIF)

S2 Fig Gating strategy for flow cytometry with the dual reporter *msnCherry*,*eaterGFP*. **(A)** Scatterplot of FSC-A against SSC-A on a logarithmical scale. Hemocytes (red dashed ellipsoid) can be readily distinguished from debris. **(B)** Overlay histograms of cells containing neither of the fluorophores (black line and black arrows), *eaterGFP*-only (green line and green arrow), and *msnCherry*-only (red line and red arrows) hemocytes. Fluorescent spillover of the GFP signal into the mCherry detector was corrected by subtracting 8.5% of the GFP signal. Non-fluorescent hemocytes were detected at low fluorescent intensity that was attributed to autofluorescense. *EaterGFP* had a one maximum peak, whereas *msnCherry* had two peaks. The fluorescent maximum from 10^6^ to 10^7^ were lamellocytes, the lower intensity peak represented *eaterGFP* and *msnCherry* double positive cell populations. **(C)** Gating strategy with intensities of cell types based on the *eaterGFP* and *msnCherry* expression. The gating strategy was worked out by the expression pattern of the dual reporter construct in blood cells of infected and age-matched control larvae was followed every second hour during a time course of 50 h after infection with *L*. *boulardi G486*. We identified five separate cell populations with varying GFP and mCherry expression and a non-fluorescent negative population. In order to reduce complexity in the current study, we grouped GFP^++^mCherry^−−^, GFP^+-^mCherry^−−^and GFP^++^mCherry^+-^ as plasmatocytes and GFP^+-^mCherry^+-^, and GFP^−−^mCherry^++^ as lamellocytes. All cells grouped as plasmatocytes had plasmatocyte morphology and expressed the plasmatocyte marker *eaterGFP*. Lamellocytes had lamellocyte morphology and expressed *msnCherry*. The dashed lines illustrate the fluorescent intensities of the five distinct blood cell populations. **(D-D”)** Hemocytes grouped as plasmatocytes had plasmatocyte morphology and expressed *eaterGFP* (D, white arrowheads) and *msnCherry* in small granules (D’, D”, red arrowheads). Hemocytes grouped as lamellocytes were large and irregularly shaped and expressed *msnCherry* (D”, yellow stars), but also had residual expression of *eaterGFP* (D’, white stars). The same representative images are shown in Fig 3A–3B’. Scale bars are 10 μm.
(TIF)

S3 Fig Overexpression of *edin* expression in the fat body is not sufficient to release the sessile hemocytes. The *in vivo* phenotype of *edin* overexpression larvae was studied using the *eaterGFP* (plasmatocytes) and *mCherry* (lamellocytes) reporters. **(A-B)** Uninfected larvae show an uninterrupted banding pattern formed by sessile plasmatocytes (green). S3 Fig shows representative images of at least 5 larvae and per genotype. The control in S3A is the same representative image as in Fig 5A. The *w;+;UAS-edin*,*Relish* ^*E20*^ [20] was separated on chromosome 3 and then backcrossed to *w* ^*1118*^ six times to create *w;+;UAS-edin*, which was used in the experiment presented in this figure.
(TIF)

S1 Table Quantification of α-tubulin to Phalloidin areas. (XLSX)
