# Supplementary figures and images for: Edin Expression in the Fat Body Is Required in the Defense Against Parasitic Wasps in Drosophila melanogaster
Source: PLoS Pathog. 2015 May 12;11(5):e1004895. doi: 10.1371/journal.ppat.1004895 (PMC4429011; doi:10.1371/journal.ppat.1004895)

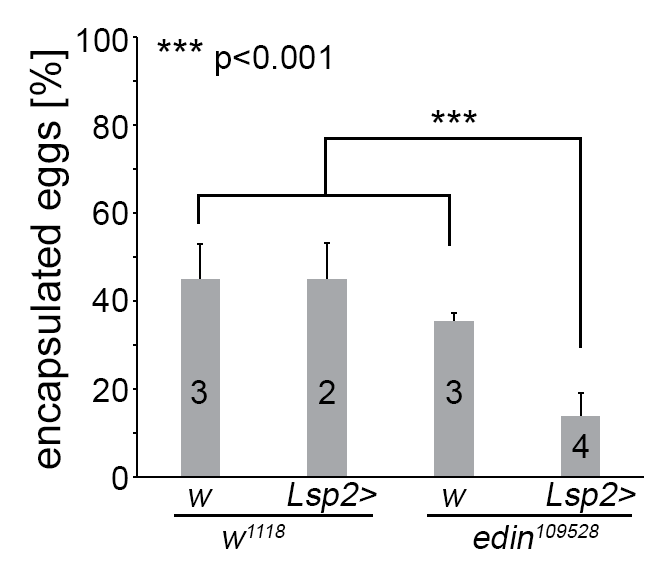

Supplement: S1 Fig — The encapsulation response of the Lsp2-GAL4-driven edin 109528 RNAi was analyzed 27-29h after a wasp infection. Data were pooled from two to four individual experiments, as depicted on each column, each experiment with at least 90 analyzed individual infected larvae. Statistical analyses were carried out as in Fig 2 using a Generalized Linear Model with binomial distribution. Error bars represent standard deviation. (TIF) [file ppat.1004895.s001.tif]

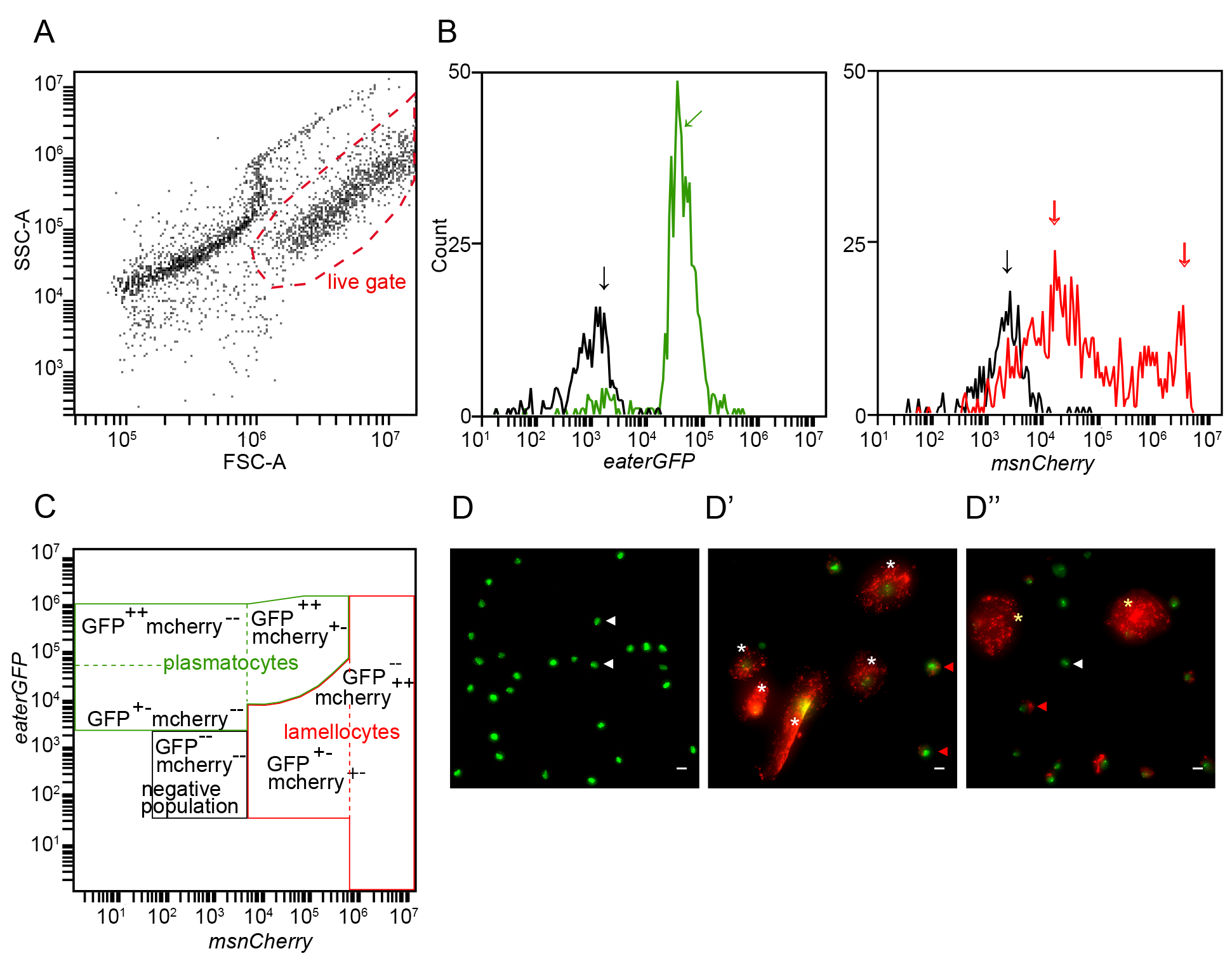

Supplement: S2 Fig — (A) Scatterplot of FSC-A against SSC-A on a logarithmical scale. Hemocytes (red dashed ellipsoid) can be readily distinguished from debris. (B) Overlay histograms of cells containing neither of the fluorophores (black line and black arrows), eaterGFP-only (green line and green arrow), and msnCherry-only (red line and red arrows) hemocytes. Fluorescent spillover of the GFP signal into the mCherry detector was corrected by subtracting 8.5% of the GFP signal. Non-fluorescent hemocytes were detected at low fluorescent intensity that was attributed to autofluorescense. EaterGFP had a one maximum peak, whereas msnCherry had two peaks. The fluorescent maximum from 106 to 107 were lamellocytes, the lower intensity peak represented eaterGFP and msnCherry double positive cell populations. (C) Gating strategy with intensities of cell types based on the eaterGFP and msnCherry expression. The gating strategy was worked out by the expression pattern of the dual reporter construct in blood cells of infected and age-matched control larvae was followed every second hour during a time course of 50 h after infection with L. boulardi G486. We identified five separate cell populations with varying GFP and mCherry expression and a non-fluorescent negative population. In order to reduce complexity in the current study, we grouped GFP++mCherry−−, GFP+-mCherry−−and GFP++mCherry+- as plasmatocytes and GFP+-mCherry+-, and GFP−−mCherry++ as lamellocytes. All cells grouped as plasmatocytes had plasmatocyte morphology and expressed the plasmatocyte marker eaterGFP. Lamellocytes had lamellocyte morphology and expressed msnCherry. The dashed lines illustrate the fluorescent intensities of the five distinct blood cell populations. (D-D”) Hemocytes grouped as plasmatocytes had plasmatocyte morphology and expressed eaterGFP (D, white arrowheads) and msnCherry in small granules (D’, D”, red arrowheads). Hemocytes grouped as lamellocytes were large and irregularly shaped and expressed msnCherry (D”, ye [file ppat.1004895.s002.tif]

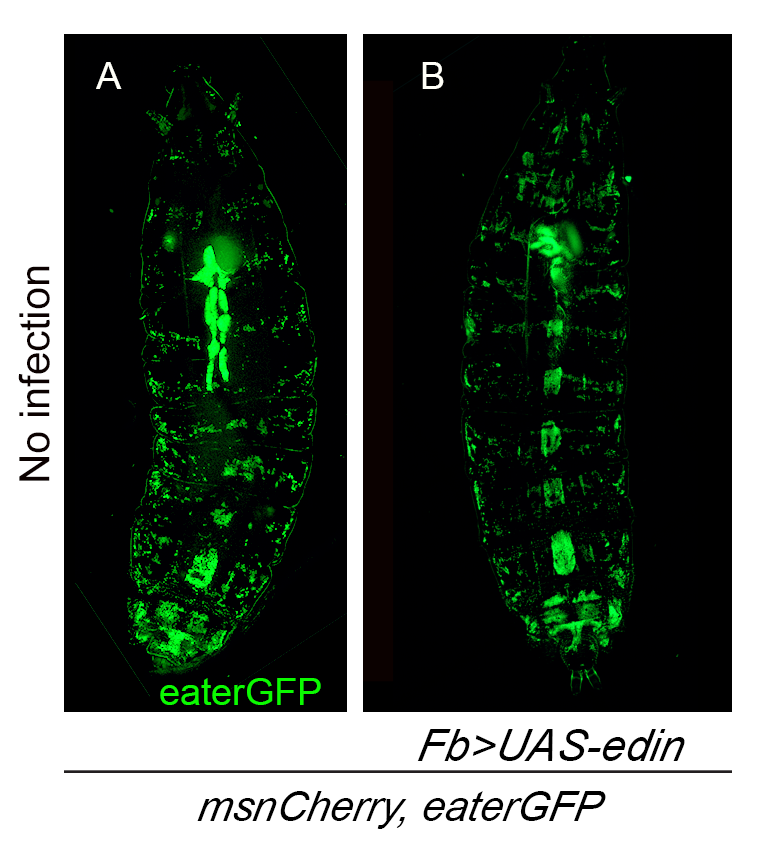

Supplement: S3 Fig — The in vivo phenotype of edin overexpression larvae was studied using the eaterGFP (plasmatocytes) and mCherry (lamellocytes) reporters. (A-B) Uninfected larvae show an uninterrupted banding pattern formed by sessile plasmatocytes (green). S3 Fig shows representative images of at least 5 larvae and per genotype. The control in S3A is the same representative image as in Fig 5A. The w;+;UAS-edin,Relish E20 [20] was separated on chromosome 3 and then backcrossed to w 1118 six times to create w;+;UAS-edin, which was used in the experiment presented in this figure. (TIF) [file ppat.1004895.s003.tif]
